# Rehabilitation after Amputation: Psychotherapeutic Intervention Module in Indian Scenario

**DOI:** 10.1155/2014/469385

**Published:** 2014-01-12

**Authors:** Kalpana Srivastava, Suprakash Chaudhury

**Affiliations:** ^1^Department of Psychiatry, Armed Forces Medical College, Pune, Maharashtra 411040, India; ^2^Department of Psychiatry, Pravara Institute of Medical Sciences (Deemed University), Rural Medical College, Ahmednagar, Loni Maharashtra 413736, India

## Abstract

Psychological aspects of adjustment to amputation are varied and not addressed in the present treatment regime. There is no research evidence available of psychological intervention and outcome in Indian scenario. One hundred and seventy-three consecutive patients with limb amputations were randomly assigned to psychotherapeutic intervention module (PIM, study group) (*n* = 90) and treatment as usual group (TAU, control group) (*n* = 83). Patients with psychotic disorder were excluded from the study. Carroll Rating Scale for Depression (CRSD), State-Trait Anxiety Inventory (STAI), Amputees Body Image Scale (ABIS), and Impact of Event Scale (IES) along with specially designed information schedule were administered individually. Structured psychotherapeutic module was developed for the intervention. Patients in PIM group were given six therapy sessions, addressing the specific areas of concern. All patients were evaluated on the same tools after two months of therapy. Analysis showed that after treatment a significant reduction in scores was noted on CRSD, STAI, ABIS, and IES in the PIM group. On the TAU group a significant reduction was seen only in the ABIS. The psychological intervention module proposed by authors was efficacious in alleviating the psychological distress, depression, and anxiety and thus was vastly superior to the conventional method of management of amputees.

## 1. Introduction

Amputation of a limb affects almost all aspects of an individual's life. Amputees in addition to their physical disability suffer from myriads of psychological as well as psychosocial problems [1]. There is little attention given on the psychological state of the individual unless he or she presents with overt behavioral abnormalities. Early recognition and treatment of psychological morbidity seem to be important in preventing long-term disabilities in an amputee. Cave paintings in Spain and France, about 36,000 years old, have shown imprints of a mutilated hand. Such paintings are also seen in New Mexico and suggest practice of self-mutilation to appease Gods in religious ceremonies [2]. Rig-Veda, an ancient sacred Indian poem, is said to have first written record of prosthesis. Written in Sanskrit between 3500 and 1800 BC, it recounts story of a warrior, Queen Vishpla, who was fitted with iron prosthesis after losing her leg in battle and returned to battle [3, 4]. Amputation has been practiced for ritualistic, punitive, curative, or vocational reasons since 43,000 BCE. Fitting with prostheses made of fiber, wood, bone, and metals, often lined with rags, has been practiced since at least 1,500 BCE [5]. The evolution of amputation as a successful technique in the treatment of injuries in World War I resulted in the first large group of amputees in history. From the time of surgery until return to normal life in the community, the majority of amputees are beset in many doubts and fears. The amputee most often grieves for the lost limb and the old body image and is thought to go through four or five stages as a part of their grieving process, that is, denial, anger, bargaining, depression, and acceptance. This often resembles the way in which people usually respond to the death of a loved one or when being diagnosed with a life threatening illness [6]. Intervention in the amputee's distress addresses the psychological side of injury and healing which is paramount to physical rehabilitation. Investigators have noted high prevalence of depressive and anxiety symptoms in amputees [7–9].

Amputation causes threefold loss in terms of function, sensation, and body image. Body image has a direct connection with psychological adjustment [10]. Body image is the scheme of our own body, which we form in our minds. It is a dynamic construction subject to revision and reconstruction in response to both internal and external stimuli. Readjusting to life after amputation is associated with reports of depression, anxiety, and disturbed body image [11]. Patients undergoing amputation as a result of traumatic injury may experience posttraumatic stress disorder. The loss of limb may confound and interact with the psychological sequel of experiences. There have been attempts to identify such problems; however psychological intervention for problems such as depression, anxiety, and social isolation has not been instituted. The occurrence of new onset of physical disability presents the patient with not only physiological, but also a psychological emergency. Role of psychological intervention will not only help in adaptation but will also enhance the well being in cases with amputation [12]. From the time of surgery until return to normal life in the community, the majority of amputees are beset in many doubts and fears. Most amputees do have the need for reassurance and constructive advice, but because amputation is a visible disability, there is usually hesitancy on the part of others to consider amputees as normal healthy individuals.

The model of therapy proposed is based on conceptual framework. Though rehabilitation is a holistic process comprising of healing, use of prosthesis, reemployment, and reintegration into the social roles, the author during her interaction with the amputees noticed certain stages of recovery process. The present study is an attempt to identify depression, anxiety, and body image disturbances and propose a model of psychological therapy in amputees. There is no research evidence available in the area of therapy in Indian setting. In fact no specific therapy module exists for these patients. Hence importance of this cannot be overemphasized. Since aim of therapy with amputees is reintegration in the social roles, the same can be achieved by targeting the concerns of amputees. These objectives were laid down in a well-defined structured module. Psychological intervention with amputees requires structured and specified areas of intervention. In view of the above present study was planned to assess psychological distress and propose a module of psychological intervention among amputees.

## 2. Material and Method 

This study was conducted at a large teaching hospital and tertiary care center for the security forces, during the period of March 2001 and August 2004. The project was approved by the institutional ethical committee. The sample is comprised of consecutive patients with recent amputations admitted during the period of study for fitting of prosthesis. Patients with major psychiatric disorder were excluded from the study. All patients were randomly assigned to either the psychotherapeutic intervention module (PIM) group or treatment as usual (TAU) group. Informed consent was obtained from all the participants of the study. Specially designed proforma was filled and all patients with amputations were interviewed during the evaluation for prosthesis and monitored over a period of two months. The following self-rating questionnaires were completed by the patients individually before and after therapeutic intervention module by a psychiatric trained nurse:

1. Carroll Rating Scale for Depression [13];
2. State-Trait Anxiety Inventory [14];
3. Body Image Scale [10];
4. Impact of Event Scale [15].

Patients in the TAU group were given one session of counseling. Patients in the PIM group were provided with psychopharmacological and psychotherapeutic treatment for a period of two months. The structured therapeutic module administered to the PIM group is comprised of following stages.

*(1) Reassurance.* Amputation can be very stressful for the amputee as well as family and friends. There is often a great deal of a free-floating anxiety about the unknown, what the future holds. Psychological support with entire gamut of coping mechanisms should be provided in a phased manner. Verbal and nonverbal assurance by the treating physician and the staff can help in adaptation to the disability.

*(2) Ventilation*. The grief response to limb loss is a complex and highly individualized one. The recognition that familiar function may not be easily reproduced may activate or intensify the grief process of the loss of limb. Simple mechanism of listening to the problems provides safety valve experience to the amputee's ventilation as a continuous release valve. This mechanism needs to be clarified to staff and family members who often try to persuade the person not to talk about the injury for fear that person will immerse himself in self-pity.

*(3) Acceptance of Self*. The process of healing requires mental work. Grief work is an active process. The main task in grief work is updating our world image. World image is like a mental map. This map represents our self-image and everything else we perceive. The real world changes significantly with the major loss; we must update our mental maps to correspond to reality. This stage is unique and poses challenges to adapt with amputation. Reorientation to the world image and correction of self-image become primarily important in amputees. Hence the reality orientation and acceptance of self can be taught at this stage which would help in adaptation to the limb loss.

*(4) Therapeutic Milieu.* Universalisation of the experience that others are also suffering has charismatic impact. It helps in the process of grieving. The stress of being alone is reduced further after coming in contact with the other amputees. It has a therapeutic impact very similar to group therapy process.

*(5) Reintegration*. In the supervised hospital environment patient develops a sense of mastery over the new patterns of physical functioning required by the limb loss. Simultaneously, a sense of emotional competence emerges as well. This learning is required to be translated into the larger perspective of social integration, so that amputees can adapt to real life challenges. Part of medical care must reinforce the person's incorporation of the hospitalization as a connecting link to life in the outside world. This can be enhanced by maintaining contact with the outside world.

The above-mentioned therapeutic module was initially tried out in an uncontrolled pilot study and found to be useful [16]. Each individual was subjected to all the phases of therapy on weekly basis, therapy was given for six sessions, and patients were reevaluated after a period of two months. At the end of therapeutic treatment, the patients were reevaluated using the same psychological tests to assess the efficacy of the treatment. *Z*-test and two-tailed “*t-*” test were applied for evaluating outcome and comparing the scores before and after treatment on psychological test.

## 3. Results

A total of 90 patients were allocated to the PIM group and 83 to the TAU group. The mean (SD) age of the PIM and TAU group patients was 30.05 (8.43) and 29.79 (8.11) years, respectively. The difference was not statistically significant. The age of the patients ranged from 22 years to 52 years. Demographic variables of the patients are given in Table 1. All the patients were males belonging to the security forces. The majority of the patients were Hindu, married, in the third decade, hailing from a rural background, and educated between 6 to 10 class. All the patients described their interpersonal relation with their family members as cordial. All the patients completed the treatment as per their group and there were no dropouts.

The scores obtained by the PIM and TAU (control group) patients on the psychological tests initially, and at the end of the study are given in Tables 2, 3, 4, 5, and 6. Analysis revealed that after treatment, there was a significant reduction in scores the Carroll Rating Scale for Depression (Table 2). Findings also revealed a significant reduction in state but not trait anxiety in the group which was given psychological intervention but not in the treatment as usual group (Tables 3 and 4). Significant reduction was noted on scores of Body Image Scale (Table 5) and Impact of Event Scale (Table 6) in the PIM group. On the TAU group, a significant reduction was only seen in the Body Image Scale (Table 5). This clearly proves the efficacy of the psychotherapeutic intervention module in amputees.

## 4. Discussion

Readjusting to life after amputation is challenging. Psychological research on the sequel of amputation has adopted an exclusive focus on the negative impact the event has on person's life. However, in the present study, an attempt has been made to formulate a structured therapeutic module of intervention in respect of amputees.

In the present study, 173 amputees were studied for a period of two months. The finding of the present study indicates the effectiveness and usefulness of psychotherapeutic intervention in ameliorating the psychological distress of the amputees. This is in agreement with the earlier studies [8, 17].

On the Carroll Rating Scale of Depression, in the PIM group, initial scores were 16.08. Therapeutic intervention reduced the score to 10.19. On the other hand, in the TAU group there was reduction of CRSD scores from 15.87 to 14.39 (differences not statistically significant). This finding again clearly emphasizes the superiority of the psychotherapeutic intervention module to the conventional management procedures.

Similarly, anxiety on initial assessment was noted to be higher which reduced significantly after therapy only in the PIM group but not in the TAU group (Table 3). Earlier studies have emphasized the need for structured therapeutic intervention for problems such as depression, anxiety, and adjustment problems [12]. Initially, psychological first aid is provided in the form of reassurance. Since amputees are required to traverse the path through denial, grieving, working through, and finally reintegrating in the midst of tragedy the parallels are drawn between loss of limb and loss of near and dear ones. During the therapy session, the emphasis was given to grieving process thereby helping in ventilation. Features of anxiety and depression were taken care of by introducing reassurance followed by ventilation. It is of relevance to emphasize that lack of communication with surgeons is a universal problem. It does not mean being insensitive to the needs of patients; rather sensitization is required in the areas of psychological aspects of amputation [18].

In the present study, body image disturbances before therapeutic intervention were significantly higher as compared to posttherapeutic intervention (Table 5). The experience of amputation introduces disruption of body image that is subsequently associated with varying degrees of body image alteration. Reconceptualisation of body image after amputation requires the incorporation of both the loss of the limb as well as incorporation of prosthesis and the crutches into the body [19]. This has recently witnessed resurgence of interest, especially among the therapists. The amputees have altered perceptions of self, as a consequence of which they consider themselves less acceptable. The prerequisite to successful adaptation is the incorporation of the prosthesis into his or her body image and focus on the future instead of lost part [1]. The associated phase of therapy is “updating the mental map.” Map making can facilitate the process of healing. Modifying body image disturbances at this stage is the main task, which is handled by embodiment of self. The process of therapy emphasized on self-acceptance and incorporation of prosthesis as a part of self. Nicholas and Robinson [18] found that acceptance of prosthesis and use of it reduce depression and help in adaptation. The emphasis during therapy was given on altered self-perception and acceptance of self.

The score on impact of event scale was higher before therapy and significant reduction was noted after therapy in the PIM group but not in the TAU group (Table 6). Patients undergoing amputation as a result of traumatic injury especially as a result of mine blast and motor vehicle accidents may also experience stress. Further, in the case of amputation, the traumatic stressor may not be temporarily delineated but rather experienced across time. The content of nightmares usually deals with the reliving of the onset of injury, future disfigurement, and concerns about disability [1]. Talking it out, analysing the fears is often useful in such instances. Reassurance along with ventilation helps in reducing the stress. In the present study, attempt was made to address the concerns of amputees during systematic phases. Peer group interactions have cathartic effect [20]. This helps in universalizing the experience. At this juncture, structured support system buffers the anxiety. This finding can be qualified by further observation, during the hospitalization; mastery of day-to-day routine is acquired through supervision and monitoring. However, these acquired skills are yet to be tested in real life settings. The final objective of the therapy is the reintegration into the social role. Acceptance of self and adaptation with the limitation of being an amputee bring badge of honor to the patient. It represents added dimension in life and brings out qualities which otherwise may have lain dormant.

## 5. Limitations

All the subjects were males due to the hospital catering to security forces. Another limitation was that patients in the TAU group were given one counseling session as per the practice, while the PIM group received more sessions of therapy.

## 6. Conclusion

In the Indian scenario, this is the first study in the domain of psychological intervention after amputation. It highlights the role of mental health professionals in the management of patients after amputation. The psychological intervention module proposed is brief and addresses highly specific concerns after amputation. In view of the high levels of anxiety and depression and body image disturbances in amputees, it is suggested that psychological evaluation and psychological intervention module should form a part of the overall management of amputees. The psychological intervention will greatly improve rehabilitation after amputation.

## Figures and Tables

**Table 1:** Demographic characteristics of the amputees.

Characteristics	PIM group (*n* = 90)	TAU group (*n* = 83)	Chi-square
Age distribution			
20–29 years	59	57	
30–39 years	21	19	*P* > 0.05
40–49 years	8	6	NS
50–59 years	2	1	
Religion			
Hindu	79	73	
Sikh	8	9	*P* > 0.5
Muslim	3	1	NS
Marital status			
Married	62	52	*P* > 0.5
Unmarried	28	31	NS
Domicile			
Rural	76	74	*P* > 0.05
Urban	14	9	NS
Education			
6–10 class	68	62	*P* > 0.05
11+	22	21	NS

S: significant; NS: not significant.

**Table 2:** Carroll Rating Scale for Depression before and after psychological intervention.

Groups	Baseline assessment mean (SD)	Assessment after intervention mean (SD)	Within group difference paired *t*-test
Study group (PIM)	16.08 (8.62)	10.19 (4.51)	*P* < 0.05
Control group (TAU)	15.87 (8.13)	14.39 (6.74)	*P* > 0.05
Between group difference *Z*-test	*P* > 0.05	*P* < 0.05	

PIM: psychotherapeutic intervention module; TAU: treatment as usual group.

**Table 3:** State-Trait Anxiety Inventory before and after psychological intervention.

Groups	Baseline assessment mean (SD)	Assessment after intervention mean (SD)	Within group difference paired *t*-test
Study group (PIM)	42.26 (7.12)	35.12 (6.46)	*P* < 0.05
Control group (TAU)	42.38 (7.09)	40.16 (6.94)	*P* > 0.05
Between group difference *Z*-test	*P* > 0.05	*P* < 0.01	

PIM: psychotherapeutic intervention module; TAU: treatment as usual group.

**Table 4:** State-Trait Anxiety Inventory before and after psychological intervention.

Groups	Baseline assessment mean (SD)	Assessment after intervention mean (SD)	Within group difference paired *t*-test
Study group (PIM)	38.68 (9.43)	33.36 (7.88)	*P* > 0.05
Control group (TAU)	39.13 (9.14)	38.07 (8.76)	*P* > 0.05
Between group difference *Z*-test	*P* > 0.05	*P* > 0.05	

PIM: psychotherapeutic intervention module; TAU: treatment as usual group.

**Table 5:** Amputees Body Image Scale before and after psychological intervention.

Groups	Baseline assessment mean (SD)	Assessment after intervention mean (SD)	Within group difference paired *t*-test
Study group (PIM)	47.21 (14.89)	29.53 (6.93)	*P* < 0.05 S
Control group (TAU)	47.68 (14.38)	38.19 (7.25)	*P* < 0.05 S
Between group difference *Z*-test	*P* > 0.05 NS	*P* < 0.05 S	

S: significant; NS: not significant; PIM: psychotherapeutic intervention module.

TAU: treatment as usual group.

**Table 6:** Impact of event scale before and after psychological intervention.

Groups	Baseline assessment mean (SD)	Assessment after intervention mean (SD)	Within group difference paired *t*-test
Study group (PIM)	37.26 (13.61)	23.44 (9.31)	*P* < 0.05 S
Control group (TAU)	37.11 (13.27)	35.87 (12.83)	*P* > 0.05 NS
Between group difference *Z*-test	*P* > 0.05 NS	*P* < 0.05 S	

S: significant; NS: not significant; PIM: psychotherapeutic intervention module.

TAU: treatment as usual group.
